# Effects of respiratory muscle training on respiratory function and functional outcomes in patients with myasthenia gravis: a systematic review

**DOI:** 10.3389/fneur.2025.1667400

**Published:** 2025-09-25

**Authors:** Zi-Ting Bi, Jing-Hua Xiao, Jing-Xue Wei, Lang Huang, Ying-Dong Li, Yue-Mi Zhang, Jian Huang, Chao-Song Luo, Jia-Mei Zhang, Yun-Shan Zhang

**Affiliations:** ^1^Department of Rehabilitation Medicine, The First Affiliated Hospital of Guangxi Medical University, Nanning, China; ^2^Department of Rehabilitation Medicine, The Second Affiliated Hospital of Guangxi Medical University, Nanning, China; ^3^Department of Rehabilitation Medicine, The Guangxi Zhuang Autonomous Region Workers’ Hospital, Nanning, China; ^4^Cardiopulmonary Rehabilitation Center, Jiangbin Hospital of Guangxi Zhuang Autonomous Region, Nanning, China; ^5^Department of Rehabilitation Medicine, Guangxi International Zhuang Medicine Hospital, Nanning, China

**Keywords:** respiratory muscle training, myasthenia gravis, respiratory function, functional outcomes, respiratory muscle strength

## Abstract

**Background:**

Respiratory muscle training is a structured intervention designed to enhance respiratory muscle function, but robust evidence on its effects in myasthenia gravis remains limited. This systematic review evaluates the impact of respiratory muscle training on respiratory function and functional outcomes in patients with myasthenia gravis.

**Methods:**

A comprehensive search of six databases was conducted without date restrictions until May 1, 2025, to identify studies meeting inclusion criteria: (1) myasthenia gravis patients aged ≥18 years, (2) respiratory muscle training involving inspiratory and expiratory muscle training, (3) outcomes on respiratory muscle strength, respiratory muscle endurance, pulmonary function tests, and functional outcome, (4) study designs like randomised controlled trials (RCTs), cohort studies, case–control trials, and quasi-experimental studies. Two reviewers independently screened studies, extracted data, and assessed methodological quality and evidence level using appropriate tools. Due to limited RCTs and heterogeneity in participants, interventions, and outcomes, a descriptive synthesis was performed.

**Results:**

Seven studies involving 223 participants (99 males and 124 females) with a mean age of 57.5 years were systematically reviewed, including two RCTs, one quasi-controlled study, one case–control study, and three cohort studies, all of which demonstrated moderate-to-high methodological quality (evidence levels 2–4). Respiratory muscle training programs involved inspiratory-expiratory training and inspiratory-only training, with parameters varying widely: intensity ranged from 15 to 75% of maximal respiratory pressures or 50 to 60% of maximal voluntary ventilation, frequency spanned from 3 to 10 sessions weekly, sessions lasted 10 to 30 min, and total intervention periods extended from 4 weeks to 12 months. Devices included threshold and variable resistance trainers, all applied under supervised conditions alongside conventional myasthenia gravis medications. All five studies evaluating respiratory muscle endurance and functional outcomes reported statistically significant enhancements (*p* < 0.05). Respiratory muscle strength and pulmonary function results were inconsistent. Maximal inspiratory pressure improved significantly in two of six studies (*p* < 0.01), while maximal expiratory pressure improved in two of three studies (*p* < 0.05). Among six studies measuring forced expiratory volume in 1 s, three measuring forced vital capacity, and three measuring peak expiratory flow, only two studies reported significant improvements in forced expiratory volume in 1 s and forced vital capacity (*p* < 0.05), while others found no effects. Adverse events reported in three studies were attributed to comorbidities rather than interventions.

**Conclusion:**

Respiratory muscle training can enhance respiratory muscle endurance and functional outcomes in patients with myasthenia gravis. However, evidence regarding its effects on respiratory muscle strength and pulmonary function remains inconsistent and is constrained by methodological limitations. Higher-quality trials are warranted to validate these findings and optimize intervention protocols.

**Systematic review registration:**

CRD42024516112.

## 1 Introduction

Myasthenia gravis (MG) is an acquired autoimmune disease, which is mediated by acetylcholine receptor antibody, cell-mediated immune dependence, and complement participation, involving the postsynaptic membrane of the neuromuscular junction, causing neuromuscular junction transmission disorder (1, 2). The clinical manifestations of this disease are fatigue and muscle weakness of skeletal muscles, which are characterized by lightness in the morning and heaviness in the evening, and temporary relief after rest (1, 3). Globally, over 700,000 individuals are afflicted with MG (4, 5). The annual incidence of MG is reported to be between 8 to 10 cases for every 1 million people, while its prevalence ranges from 150 to 250 cases per 1 million individuals (4, 5). MG affects all age groups, with the most common age of onset being 20–39 years in women and 50–70 years in men (6). As the population ages, patients over the age of 65 are gradually increasing among MG patients (4, 7). MG has a wide range of impacts on patients’ physical, psychological and social health, resulting in reduced health-related quality of life, which can bring huge psychological and economic burdens to themselves and their families (8). Therefore, it is necessary to pay special attention to the clinical treatment and rehabilitation management of MG patients.

The muscles affected in MG are skeletal muscles, and the earliest affected muscles are mostly extraocular muscles (1). As the disease progresses, it can gradually affect muscle groups like limb muscles, throat muscles, and respiratory muscles, resulting in a typical pattern of muscle weakness (9, 10). This pattern of muscle weakness in MG is characterized by a gradual decline in respiratory muscle strength and endurance (11), and its clinical manifestations are rapid and shallow breathing patterns at rest, prone to upper airway obstruction, sleep apnea, and even respiratory failure, which severely limits the activities of daily living (12, 13). Although objective indicators can show that the vital capacity of the lung function of patients with MG is normal, the respiratory capacity decreases during the maximum spontaneous ventilation period, and the respiratory muscle strength and endurance decrease, resulting in respiratory dysfunction (11, 14, 15). Respiratory muscle dysfunction can further worsen the patient’s physical health, limit motor function, affect quality of life, and even increase the risk of MG crisis (8, 9, 13, 16). MG crisis is a life-threatening complication that occurs suddenly during the onset or treatment of MG, and endotracheal intubation and mechanical ventilation are required in severe cases (17, 18). Additionally, coupled with the influence of limb muscle involvement on limb muscle strength, patients with MG often have motor dysfunction, which can further deteriorate the overall physical health of the patient, and in turn this may indirectly reduce respiratory function (8, 9, 19, 20). Therefore, it is of great clinical significance to actively study effective treatments for improving respiratory dysfunction in patients with MG.

The clinical treatment of MG mainly includes cholinesterase inhibitors, immunosuppressive agents, adrenal cortical hormone, immunoglobulin, plasma exchange and thymectomy (21–24). Though the timely intervention of clinical treatment has improved the survival rate of patients with MG, they may face residual complications and dysfunction (25, 26). Patients with MG may often have respiratory dysfunction due to myasthenic symptoms and fatigue, which limits motor function and reduces the quality of life (8, 27, 28). Therefore, measures to intervene in these symptoms and dysfunction are very necessary. Studies have suggested that rehabilitation for MG is an important auxiliary means in addition to drug therapy and surgical treatment (1, 29, 30), and may become an effective intervention to improve complications, dysfunction, and quality of life in patients with MG (8, 31, 32). In addition to routine rehabilitation, respiratory muscle training (RMT) may be helpful for patients with MG. RMT is a therapeutic approach that utilizes a portable device to deliver regulated training to the respiratory muscles by imposing pressure thresholds or flow-dependent resistance during inhalation or exhalation, stimulating the respiratory muscles to respond and produce changes in muscle structure, thereby enhancing the strength and endurance of the respiratory muscles and improving respiratory function (33). Although preliminary clinical studies (34, 35) suggest that RMT may be beneficial to the prognosis of patients with MG, there is still a serious lack of high-quality evidence to prove the effectiveness of RMT in patients with MG. Therefore, it is necessary to systematically review the clinical application of RMT in patients with MG, so as to explore its effectiveness.

At present, there is only one review (36) published in 2009 that separately sorts out the evidence of RMT for patients with MG, but no systematic review was found on the effect of RMT on respiratory function and functional prognosis in patients with MG. Although this review supports the use of RMT in patients with MG, only three studies were included in this review. Few participants were included in this review, and one of the studies included patients with three different neuromuscular diseases. Since the publication of this review in 2009, many studies aimed at analysing the effects of RMT on respiratory function and functional capacity in patients with MG have been published. Therefore, a systematic review in this field is necessary.

Thus, the objectives of this systematic review were to synthesize the existing evidence on RMT for patients with MG, so as to explore the effects of RMT on respiratory and functional outcomes in patients with MG, and provide clinical practice guidelines for the rehabilitation of these patients.

## 2 Materials and methods

This systematic review was conducted in alignment with the PRISMA guidelines, which delineate the essential reporting elements for executing a systematic review (37).

### 2.1 Eligibility criteria

The criteria for inclusion were established based on the Population-Interventions-Comparison-Outcomes of interest-Study design (PICOS) framework (38), as outlined in Table 1. The exclusion criteria were: (1) abstracts, letters, case reports, reviews, protocol, or unusable full text; (2) MG patients with perioperative period of thymectomy; (3) inadequate intervention strategies arise from the ambiguous characterization of the training program concerning its intensity, duration, and frequency; (4) research that fails to disclose the noteworthy outcome variables.

**Table 1:** Inclusion criteria.

Population: MG patients with age ≥18 years old.
Intervention: Respiratory muscle training includes inspiratory and expiratory muscle training.
Control: Sham respiratory muscle training or a rehabilitation program that does not incorporate respiratory muscle training.
Outcomes: 1. Respiratory function: respiratory muscle strength (MIP, MEP), respiratory muscle endurance, and pulmonary function tests (PEF, FEV1, FVC). 2. Functional outcome: QMG score, MGC scale, MG score, and ADL.
Study Design: RCTs, cohort studies, case–control trials, and quasi-controlled study.

MG, Myasthenia Gravis; RCT, Randomised Controlled Trial; MIP, Maximal Inspiratory Pressure; MEP, Maximal Expiratory Pressure; PEF, Peak Expiratory Flow; FEV1, Forced Expiratory Volume in 1 s; FVC, Forced Vital Capacity; QMG, Quantitative Myasthenia Gravis; MGC, Myasthenia Gravis Composite; ADL, Daily Living Ability.

### 2.2 Information sources

The systematic search was conducted in six databases (PubMed, Embase, Allied and Complementary Medicine Database, Cumulative Index to Nursing and Allied Health Literature, Cochrane Library, and China National Knowledge Infrastructure databases) without date limits up to May 01, 2025.

### 2.3 Search strategy

To ensure the relevance of the retrieved documents to the thematic focus, a comprehensive search was conducted in related electronic databases utilizing keywords and associated terms, combined with Boolean operators and truncations, while imposing no restrictions on language. An exhaustive and systematic approach for literature retrieval was developed, as detailed below. To mitigate potential bias, following the initial search of the primary database, an additional manual search was conducted utilizing the reference lists of all selected articles and reviews to guarantee the thorough collection of relevant literature. The specific search processes of all databases are shown in Appendix 1.

(“Myasthenia Gravis” OR “MG” OR “Generalized Myasthenia Gravis”) AND (“respiratory strength training” OR “inspiratory strength training” OR “expiratory strength training” OR “respiratory muscle training” OR “RMT” OR “inspiratory muscle training” OR “IMT” OR “expiratory muscle training” OR “EMT” OR “breathing muscle training” OR “breathing exercises”) AND (“respiratory function” OR “respiratory muscle strength” OR “maximum inspiratory pressure” OR “MIP” OR “maximum expiratory pressure” OR “MEP” OR “respiratory muscle endurance” OR “pulmonary function tests” OR “peak expiratory flow” OR “PEF” OR “forced expiratory volume in 1 s” OR “FEV1” OR “forced vital capacity” OR “FVC” OR “functional outcome” OR “quantitative myasthenia gravis score” OR “QMG score” OR “myasthenia gravis composite scale” OR “MGC scale” OR “MG score” OR “daily living ability” OR “ADL”).

### 2.4 Selection process

The studies obtained were compiled and managed using EndNote 20 software, and duplicate studies were removed. Subsequently, two reviewers (ZT and JX) independently assessed the titles and abstracts of the studies based on the predetermined eligibility criteria for initial screening, eliminating any literature that did not satisfy the inclusion criteria. They then proceeded to examine the full texts of the remaining studies to further ascertain compliance with the inclusion criteria and to identify the specific reasons for excluding studies that did not qualify. Finally, the reviewers engaged in face-to-face discussions and proofreading to finalize the list of included studies. In instances where there was a disagreement between the two reviewers regarding the results of a study or its potential inclusion, the matter was resolved through discussion or by consulting a third reviewer (JH).

### 2.5 Data collection process

In order to minimize discrepancies and inaccuracies during the data extraction phase, two reviewers (YD and LH) independently retrieved data pertinent to the evaluation query utilizing standardized data extraction forms that were modified from the Joanna Briggs Institute (JBI) instrument. The JBI tool is recognized as an appropriate resource for extracting data across diverse research methodologies (39). Additionally, this instrument not only simplifies the data collection process but also provides well-structured data, thereby enhancing both comparison and analysis (39). In order to guarantee the identification and retrieval of pertinent data while reducing the potential for bias and errors, standard data extraction forms were evaluated before the official data extraction process. The information gathered from the selected studies encompassed the following aspects: research background (author/year/country), epidemiological constructs (study design/sample size/participant profiles), specifics of intervention (modality/intensity/frequency/duration/devices/supervision/progression), outcome metrics, results (means and *p*-values), as well as any additional relevant information by the guidelines outlined in the “Cochrane Handbook for Systematic Reviews of Interventions (40).” When some necessary information was lacking, the reviewer (YM) contacted the corresponding author of the article by telephone or e-mail to obtain the missing data. Any disagreement over data extraction content was resolved by discussion until a consensus was reached. The extracted data were verified by a third reviewer (CS).

### 2.6 Methodological quality and level of quality evidence

To reduce the risk of bias and increase the transparency and rigour of the review process, the tool chosen for the quality appraisal of this systematic review based on the types of included studies, the Physical Therapy Evidence Database (PEDro) scale for RCTs (41), the JBI Critical Appraisal Checklist for quasi-controlled studies (42), and the Newcastle-Ottawa Scale (NOS) for case–control and cohort studies (43, 44). The PEDro scale includes 11 items, and each needs to answer Yes, No (45). The total score ranges from 0 to 10 points, and higher scores indicate superior methodological quality (46). Studies with scores between 9 and 10 are considered ‘excellent’, and scores from 6 to 8 are assessed as good, whereas scores of 5 and 4 are classified as fair quality, and scores below 4 are considered as poor quality (47, 48). The JBI critical appraisal checklist includes 9 items and each needs to answer Yes, No, Unclear or Not/Applicable (42). The rating score is from 1 to 9 points, and quality scores are divided into three groups: 1 to 4 points for low quality, 5 to 7 points for medium quality, and 8 to 9 points for high quality (49). The NOS includes 3 quality parameters: 4 points for selection, 2 points for comparability, and 3 points for exposure/outcome assessment (43, 44). The total score ranges from 0 to 9 points. Studies with the NOS scores of 7 or higher are considered as ‘high-quality’, and scores of 5 to 6 are assessed as ‘moderate quality’ (44). Additionally, the level of quality evidence for the include studies was assessed using the Oxford Centre for Evidence-Based Medicine (OCEBM)-Levels of Evidence guide (50). The levels of Evidence include: level 1, systematic review of randomized trials or n-of-1 trials; level 2, randomized trial or observational study with dramatic effect; level 3, Non-randomized controlled cohort/follow-up study; level 4, Case-series, case–control studies, or historically controlled studies; level 5, Mechanism-based reasoning. Level 1 represents the strongest possible evidence, while level 5 represents the weakest possible evidence. Two reviewers (JH and YS) with the same critical evaluation knowledge level used the suitable tool according to the types of included studies and the OCEBM to independently assess methodological quality and level of quality evidence. Any disagreements with the score were resolved through discussion. If there were still any disagreements about the assessment result score between the two primary reviewers, a third reviewer (JM) would resolve them. Due to the limited number of included studies, publication bias was not evaluated.

### 2.7 Data synthesis and analysis

The extracted data was synthesized. Meta-analyses were performed only when the data for the analysed variables were at least 3 RCT studies (40). Otherwise, a narrative synthesis would be carried out within and between articles. However, due to limited RCTs in this aspect and significant clinical heterogeneity in participant demography, intervention details, and outcome collection among the included studies, it was appropriate to construct a narrative synthesis. The findings are reported as the mean with standard error or as a *p*-value.

## 3 Results

**Figure 1:**
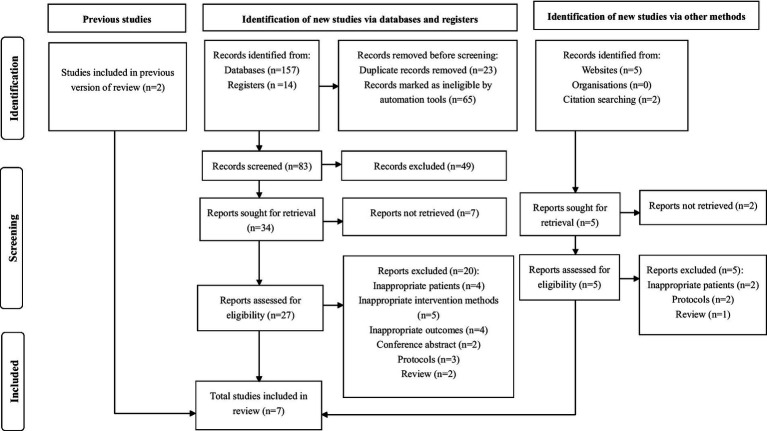
PRISMA search flow diagram.

A total of 171 potentially pertinent studies were identified from the relevant data sources, encompassing 157 studies sourced from electronic databases, 14 from registries, and 7 from websites and citation searching. Following this, 88 duplicate studies were eliminated, resulting in a total of 83 studies. Upon reviewing the titles and abstracts, 49 irrelevant articles were discarded, leaving 34 studies that necessitated obtaining and reading the full texts, but 7 studies could not be accessed. Concurrently, 7 studies were sourced from both websites and citation searching, yet 2 articles remained unattainable. Subsequently, the 32 accessible studies underwent an eligibility evaluation. Ultimately, 7 studies (9–11, 34, 35, 51, 52) that satisfied the eligibility criteria were incorporated into this review, while the remaining 25 studies were excluded for reasons like inappropriate patients, inappropriate intervention methods, or review. Figure 1 illustrates the PRISMA flow diagram outlining the study selection process.

### 3.1 Characteristics of the included studies

These 7 studies included two RCTs (51, 52), one quasi-controlled study (9), one case–control study (10), and three cohort studies (11, 34, 35). The studies were conducted between 1998 and 2020, with two articles (35, 52) included in a previous review. Three studies were conducted in Germany (10, 11, 34), two studies were performed in China (9, 51), and one study each was conducted in Spain (52) and Israel (35). Although these studies took place in different countries, the synthesis of evidence can provide help and guidance for clinical treatment and research. The main characteristics of the included studies are listed in Table 2.

**Table 2:** Characteristics of the included 7 studies.

Study	Country	Design	Participants	Intervention	Control	Main results	Key findings	Adverse events
Hsu et al. (9)	China	Quasi-controlled studies	*n* = 34 IG: 18 CG: 16 Male/female:13 /21 Mean age(y): 58.3 Mean disease duration (y): 9.2 MGFA classification: IIa-IIIb Mild to moderate MG Thymectomy (Y/N): 21/13 Medical and treatment history: cholinesterase inhibitors	Type of RMT: IMT + EMT (Details in Table 3) Conventional treatment as CG	Conventional treatment: cholinesterase inhibitors	RMT significantly increased FVC from 77:9 ± 12:6% to 83:8 ± 17:7% (*p* = 0:03) and FEV1 from 75:2 ± 18:3% to 83:3 ± 19:0% (*p* = 0:002), except for MIP and MEP. The QMG score improved from 9:6 ± 4:1 to 8:1 ± 4:3 (*p* = 0:04) and the MGC scale from 4:4 ± 3:5 to 2:7 ± 2:9 (*p* = 0:02)	RMT can improve FVC, FEV1, QMG score, and MGC scale in patients with mild to moderate generalized MG	NR
Freitag et al. (10)	Germany	Case–control	*n* = 24 IG: 18 CG: 6 Male/female: 8/16 Mean age(y): 55.3 Mean disease duration (y): 12.5 MGFA classification: IIa-IIb Mild to moderate MG Thymectomy (Y/N): 9/15 Medical and treatment history: cholinesterase Inhibitors, azathioprine, methotrexate, prednisone, immunoglobulin	Type of RMT: IMT + EMT (Details in Table 3) Conventional treatment as CG	Conventional treatment: clinical drug treatment	Thirteen months of RMT significantly increased RE measured as time until exhaustion to 412% of baseline (*p* < 0.001). The MG score improved from 0.67 ± 0.09 to 0.41 ± 0.1 (*p* = 0.004). FEV1, PEF, MIP did not change during the training period (*p* > 0.05)	Long-term RMT is a significant increase in RE, and enhanced RE was associated with significant alleviation of MG symptoms as shown by the MG score	Majority of patients experienced ≥1 interruption due to health issues (e.g., infections, trauma, surgery), often associated with transient MG deteriorations requiring medication adjustment. Interruptions and deteriorations were triggered by comorbidities, not directly by RMT
Huang (51)	China	RCT	*n* = 100 IG: 50 CG: 50 Male/female: 50 /50 Mean age(y): 65.8 Mean disease duration (y): NR MGFA classification: NR Mild to moderate MG Thymectomy (Y/N): NR Medical and treatment history: cholinesterase inhibitors	Type of RMT: IMT + EMT (Details in Table 3) Conventional treatment as CG	Conventional treatment: clinical drug treatment	The Barthel index score for ADL in IG increased from 21.3 + 2.25 to 80.12 + 1.13, and the difference between the two groups was statistically significant (*p* < 0.05)	RMT can improve ADL in patients with mild to moderate MG	N
Rassler et al. (11)	Germany	Cohort study	*n* = 10 IG: 10 Male/female: 5/5 Mean age(y): 60.4 Mean disease duration (y): 9.5 MGFA classification: IIa-IIb Mild to moderate MG Thymectomy (Y/N): NR Medical and treatment history: cholinesterase inhibitors, immunotherapy with azathioprine	Type of RMT: IMT + EMT (Details in Table 3) Clinically relevant medications	N/A	IMT + EMT improved myasthenia score from 0.71 ± 0.1 to 0.56 ± 0.1 (*p* = 0.007). Respiratory endurance time increased from 6.1 ± 0.8 to 20.3 ± 3.0 min (p < 0.001). MIP and lung function (FEV1, PEF) did not change (*p* > 0.05)	RMT improved myasthenia score and RE in patients with mild to moderate MG	One patient developed a respiratory infection during project training duration, but it was not training-induced
Rassler et al. (34)	Germany	Cohort study	*n* = 10 IG: 10 Male/female:4 /6 Mean age(y): 53 Mean disease duration (y): 5 MGFA classification: NR Mild to moderate MG Thymectomy (Y/N): NR Medical and treatment history: cholinesterase inhibitors, immunotherapy with azathioprine	Type of RMT: IMT + EMT (Details in Table 3) Clinically relevant medications	N/A	IMT + EMT significantly increased RE from 8.4 ± 0.9 min to 17.1 ± 1.3 min (*p* < 0.001). MG score, MIP and lung function (FEV1, PEF) did not change (*p* > 0.05)	RMT enhanced RE in patients with mild to moderate MG	N
Fregonezi et al. (52)	Spain	RCT	*n* = 27 IG: 14 CG: 13 Male/female:11/16 Mean age(y): 64 Mean disease duration (y): NR MGFA classification: IIa-IIb Mild to moderate MG Thymectomy (Y/N):9/18 Medical and treatment history: NR	Type of RMT: IMT (Details in Table 3) Conventional treatment as CG	Conventional treatment: pyridostigmine bromine, azathioprine, and prednisone	The IG improved significantly compared to control group in MIP, MEP, RE (respectively *p* = 0.001, =0.01, <0.05). No significant improvement was seen in lung function (FVC, FEV1) (*p* > 0.05)	RMT can improve respiratory strength and endurance in patients with mild to moderate MG	One patient experienced a myasthenic crisis during the preprogram training period, but it was not training-induced
Weiner et al. (35)	Israel	Cohort study	*n* = 18 IG1: 10 IG2: 8 Male/female: 8/10 Mean age(y): 45.9 Mean disease duration (y): NR MGFA classification: NR IG1: Mild to moderate MG IG2: severe MG Thymectomy (Y/N):13/18 Medical and treatment history: anticholinesterase, prednisone	Type of RMT: IMT + EMT(IG1), IMT(IG2). (Details in Table 3) Drug treatment like anticholinesterase and prednisone	N/A	The MIP, RE, FVC, and FEV1 increased significantly in both groups (respectively *p* < 0.001, <0.005, <0.001, <0.001, <0.001, <0.001, <0.001, <0.001). The MEP increased significantly in IG1 (*p* < 0.05) but remained unchanged in IG2	IMT alone and IMT + EMT markedly improved MIP, RE, FVC, and FEV1. Only IMT + EMT improved MEP	NR

MG, Myasthenia Gravis; RCT, Randomised Controlled Trial; IG, Intervention Group; CG, Control Group; MGFA, Myasthenia Gravis Foundation of America; RMT, Respiratory muscle training; IMT, Inspiratory muscle training; EMT, Expiratory muscle training; MIP, Maximal Inspiratory Pressure; MEP, Maximal Expiratory Pressure; RE, respiratory endurance; PEF, Peak Expiratory Flow; FEV1, Forced Expiratory Volume in 1 s; FVC, Forced Vital Capacity; QMG, Quantitative Myasthenia Gravis; MGC, Myasthenia Gravis Composite; ADL, Daily Living Ability; NR, Not reported in the source trial; Y, Yes; N, No; N/A, Not Available.

#### 3.1.1 Participants

A total of 223 participants were enrolled in the included studies, with the number of participants varying from 10 (11, 34) to 100 (51). The age distribution among participants exhibited minor variations across the different studies, with individuals’ ages spanning from 21 years (35) to 76 years (10). The mean age of the participants was 57.5 years. This reflects that the incidence of MG may tend to be younger, in addition to older age. The participants in each study comprised both males and females, with a total of 99 males and 124 females. Four investigations (9–11, 34) indicated the duration of the illness, while three others (35, 51, 52) failed to present data regarding this aspect. The average duration of the disease among the participants was 9.05 years. Concerning the Myasthenia Gravis Foundation of America (MGFA) classification, three studies (10, 11, 52) indicated that participants fell within the IIa to IIb spectrum, one study (9) categorized participants within the IIa to IIIb range, while three additional studies did not provide data pertinent to this classification (34, 35, 51). Participants in these studies included patients with mild to moderate MG. Interestingly, the study of Weiner et al. (35) contained severe MG patients in another intervention group besides an intervention of patients with mild to moderate MG. Regarding thymectomy history, four studies (9, 10, 35, 52) reported specific participant data, of which 51 had surgical resection and 64 did not, while three studies (11, 34, 51) did not provide this information. Concerning the medical treatment history, six studies (9–11, 34, 35, 51) consistently reported the use of cholinesterase inhibitors, with four of these (10, 11, 34, 35) additionally documenting various immunotherapies including azathioprine, prednisone, methotrexate, and immunoglobulin; however, one study (52) did not report any medical treatment history.

#### 3.1.2 Interventions

All included studies performed RMT, five studies (9–11, 34, 51) performed inspiratory muscle training (IMT) and expiratory muscle training (EMT), and one study (52) only carried out IMT. Interestingly, the study of Weiner et al. (35) had two intervention groups, one performed IMT and EMT, and the other only carried out IMT. The parameters of intervention in each study were variable (As shown in Table 3). The intensity of RMT in three studies (9, 35, 52) started at 15 to 60% of MIP or 15 to 75% MEP, and three studies (10, 11, 34) used 50 to 60% of individual MVV/VC and a frequency of 25–35 breaths/min. However, the study of Huang (51) did not report on the intensity of RMT. The frequency and duration of intervention were also different across the studies. The time of each treatment session varied from 10 to 30 min. Six studies were 30 min (9–11, 34, 35, 51), while only one study (52) was 10 min. Furthermore, RMT were carried out 3 to 10 times per week. In terms of the duration of the intervention, the duration of the intervention in six studies (9, 11, 34, 35, 51, 52) was between 4 weeks and 12 weeks, while only the study by Freitag et al. (10) conducted 4 weeks of intensive training and 12 months of maintenance training. Regarding the use of devices, they were different: Dofin breathing trainer (9), Threshold trainer (35, 52), Respiration training device (51), Portable device (10, 11, 34). Additionally, four studies (9, 35, 51, 52) were threshold resistance, while three studies (10, 11, 34) were variable resistance. Although different types of devices were used, these studies were strength training. All study interventions were executed under the guidance of supervision. Besides, all the included studies adjusted the intensity of the intervention accordingly. In the included studies, all patients, regardless of whether they were in the intervention or control group, received conventional clinical treatment, mainly cholinesterase inhibitors, usually in combination with other immunosuppressants like azathioprine, prednisone, methotrexate, or immunoglobulin.

**Table 3:** RMT parameters of the included 7 studies.

Study	Intensity	Frequency	Duration	Device	Supervision	Progression
Hsu et al. (9)	30 to 60% of MIP and 15 to 75% of MEP	30 min, twice a day, 5 times/week	12 weeks	The dofin breathing trainer (threshold resistance)	Supervised by the trainer	Resistance was adjusted accordingly
Freitag et al. (10)	50–60% of MVV and VC, and frequency of 25–35 breaths/min	30 min, 5 times/week	4 weeks intensive training, 12 months maintenance training	The portable device (flow resistance)	Supervised by the trainer	Resistance was adjusted accordingly
Huang (51)	NR	30 min, 5 times/week	4 weeks	Respiration training device (threshold resistance)	Supervised by the trainer	Resistance was adjusted accordingly
Rassler et al. (11)	50–60% of MVV and VC, and frequency of 25–35 breaths/min	Phase 1: 30 min, 20 training sessions, 5 times/week Phase 2: 30 min, 5 times/2 weeks	Phase 1: 4 weeks Phase 2: 3 months	The portable device (flow resistance)	Phase 1: Supervised at the laboratory by the trainerPhase 2: Supervised at home by phone	NR
Rassler et al. (34)	50–60% of MVV and VC, and frequency of 25–35 breaths/min	30 min, 20 training sessions, 5 times/week	4–6 weeks	The portable device (flow resistance)	Supervised by the trainer	NR
Fregonezi et al. (52)	20% of MIP	10 min, 3 times/week	8 weeks	Threshold IMT (threshold resistance)	Supervised by the trainer	Resistance was increased to 30% in the third week, 45% in the fifth week, and 60% in the seventh
Weiner et al. (35)	15% of MIP or 15% of MEP	30 min,6 times/week	12 weeks	Threshold Trainer (threshold resistance)	Supervised by the trainer	Resistance was increased incrementally, 5% each session, to reach 60% of their MIP or MEP at the first month and then continued for the next 2 months

RMT, Respiratory muscle training; IMT, inspiratory muscle training; MIP, Maximal Inspiratory Pressure; MEP, Maximal Expiratory Pressure; MVV, Maximal Voluntary Ventilation; VC, Vital Capacity; NR, Not reported in the source trial.

### 3.2 Effect of interventions

#### 3.2.1 Effect of RMT on respiratory muscle strength

Six studies (9–11, 34, 35, 52) showed results about MIP, and three studies (9, 35, 52) assessed MEP. An RCT conducted by Fregonezi et al. (52) showed that 8 weeks of IMT had a statistically significant improvement in MIP and MEP within and between groups in MG patients (*p* = 0.001, *p* = 0.01), indicating that IMT could improve MIP and MEP after 8 weeks of intervention. Moreover, a cohort study conducted by Weiner et al. (35) indicated that IMT + EMT increased MIP (p = 0.001) and MEP (*p* < 0.05) in patients with mild to moderate MG, while IMT alone only improved MIP (p = 0.001) in patients with severe MG. However, in a recent quasi-controlled study, Hsu et al. (9) reported that RMT did not significantly improve MIP and MEP (*p* > 0.05). Furthermore, Rassler et al. (11, 34) conducted cohort studies in 2007 and 2011, respectively. They found that RMT did not change MIP (*p* > 0.05). A case–control study conducted by Freitag et al. (10) in 2018 also found the same result. The results of these three studies suggested that RMT was not statistically significant in improving MIP (*p* > 0.05), but their studies showed that MIP and MEP were normal or slightly lower at baseline. Therefore, it is not surprising that their results found that RMT did not significantly improve MIP or MEP in patients with MG.

#### 3.2.2 Effect of RMT on respiratory muscle endurance

Five studies (10, 11, 34, 35, 52) measured the results of respiratory muscle endurance. The RCT by Fregonezi et al. (52) indicated that IMT produced statistically significant improvement in respiratory muscle endurance in patients with MG compared with the control group (*p* < 0.05). Furthermore, the case–control study by Freitag et al. (10) studied the effects of 4 weeks of intensive RMT and 12 months of maintenance RMT on MG patients and compared the results with the control group. They found that both short-term and long-term RMT could improve respiratory muscle endurance in MG patients (*p* < 0.001). Three cohort studies (11, 34, 35) also found the same result. Rassler et al. (11, 34) found that RMT could promote respiratory muscle endurance when they performed two cohort studies in 2007 and 2011 (*p* < 0.001). Similarly, Weiner et al. (35) found that IMT alone improved respiratory muscle endurance in severe MG patients (*p* < 0.001), while IMT + EMT improved respiratory muscle endurance in mild to moderate MG patients (*p* < 0.001). These studies suggest that RMT can improve respiratory muscle endurance in patients with MG.

#### 3.2.3 Effect of RMT on pulmonary function

Six studies (9–11, 34, 35, 52) assessed FEV1, three studies analysed PEF (10, 11, 34), and three studies (9, 35, 52) reported FVC. Only one quasi-controlled study (9) and one cohort study (35) found that RMT had a statistically significant improvement in FEV1 and FVC for patients with MG (*p* < 0.05). However, one RCT (52) held the opposite view that IMT did not produce statistically significant improvements in FEV1 and FVC when compared to the control group (*p* > 0.05), but the within-group comparison was statistically significant (*p* < 0.05). Additionally, one case–control study (10) and two cohort studies (11, 34) reported that RMT had no statistically significant effect on FEV1 and PEF (*p* > 0.05). It is important to note that these three studies included participants with normal or slightly low lung function at baseline, so their findings need to be considered carefully.

#### 3.2.4 Effect of RMT on functional outcomes

Five studies (9–11, 34, 51) collected functional outcome data using different evaluation forms. One RCT by Huang (51) used ADL through the Barthel index score to assess the functional outcome of RMT in patients with MG. The Barthel index score for ADL was significantly improved, and the difference between groups was statistically significant (*p* < 0.05). One quasi-controlled study by Hsu et al. (9) adopted the QMG score and MGC scale to observe the effect of RMT on functional outcome in patients with MG. This study showed that the QMG score and MGC scale of the intervention group were improved, and there was a significant difference in QMG score (*p* = 0.04) and MGC scale (*p* = 0.02) between the groups. Besides, one case–control study (10) and two cohort studies (11, 34) analysed functional outcome through MG score. Freitag et al. (10) found that long-term RMT improved MG score in patients with MG (*p* = 0.004). Rassler et al. (34) did a cohort study in 2007. This study observed that 4 to 6 weeks of RMT could improve MG score, but there was no statistically significant improvement in MG score (*p* > 0.05). However, Rassler et al. (11) increased the intervention duration based on the previous experiment in 2011, namely, the first stage was 4 weeks and the second stage was 3 months. They found that a relatively long RMT could improve MG score with statistical significance (*p* = 0.007). Although these studies used different assessment methods to analyse functional outcome, their findings suggest that RMT can promote functional outcome in patients with MG.

#### 3.2.5 Adverse events of included studies

In terms of adverse events reporting, three studies (10, 11, 52) documented relevant data, while two trials (34, 51) explicitly reported no adverse events occurred, and another two studies (9, 35) did not provide information on this outcome. Freitag et al. (10) observed that the majority of patients experienced at least one treatment interruption due to health complications (e.g., infections, trauma, or surgery), typically accompanied by transient MG deteriorations necessitating medication adjustments. Crucially, these interruptions and deteriorations were attributed to comorbidities rather than RMT itself. Similarly, Rassler et al. (11) noted one case of respiratory infection during the training period, which was unrelated to the intervention. Fregonezi et al. (52) also reported one myasthenic crisis occurring in the preprogram phase, with no causal link to training. These findings suggest that comorbidities, rather than RMT, are the primary factor contributing to clinical worsening in this patient population.

### 3.3 Methodological quality of included studies

The methodological quality was critically assessed using the PEDro scale for two RCTS, the JBI critical appraisal checklist for one quasi-experimental study, and the NOS for one case–control study and three cohort studies. Tables 4–6 shows each item’s score and the total score of the 7 included studies. The methodological quality of two RCTs by Fregonezi et al. (52) and Huang (51) was considered ‘good’ with a total score of 6 points based on the PEDro scale. The quasi-experimental study by Hsu et al. (9) was regarded as ‘high quality’ with a total score of 8 points by the JBI critical appraisal checklist. According to the NOS, the cohort study by Rassler et al. (11) in 2011 received a total score of 6 points, and the remaining studies (10, 34, 35) received a total score of 5 points, so they were evaluated as ‘moderate quality’.

**Table 4:** Quality score on the PEDro scale of two RCTs.

Study	Random allocation	Concealed allocation	Baseline similarity	Blind subjects	Blind therapists	Blind assessors	Adequate follow-up	Intention-to-threat analysis	Between-group comparisons	Point estimates and variability	Total score
Huang. (51)	Y	N	Y	N	N	N	Y	Y	Y	Y	6
Fregonezi et al. (52)	Y	N	Y	N	N	Y	Y	N	Y	Y	6

PEDro: Physiotherapy Evidence Database. 1: Y: Yes; N: No. The total score of PEDro: 10.

**Table 5:** Quality score on the JBI critical appraisal checklist of one quasi-experimental study.

Study	1	2	3	4	5	6	7	8	9	Total score
Hsu et al. (9)	Y	Y	U	Y	Y	Y	Y	Y	Y	8

JBI: Joanna Briggs Institute. The following 9 items were included: 1. Is it clear in the study what is the ‘cause’ and what is the ‘effect’ (i.e., there is no confusion about which variable comes first)? 2. Were the participants included in any comparisons similar? 3. Were the participants included in any comparisons receiving similar treatment/care, other than the exposure or intervention of interest? 4. Was there a control group? 5. Were there multiple measurements of the outcome both pre and post the intervention/exposure? 6. Was follow up complete and if not, were differences between groups in terms of their follow up adequately described and analyzed? 7. Were the outcomes of participants included in any comparisons measured in the same way? 8. Were outcomes measured in a reliable way? 9. Was appropriate statistical analysis used? Y: Yes; N: No; U: Unclear; N/A: Not Available.

**Table 6:** Quality score on the Newcastle-Ottawa Scale of one case–control study and three cohort studies.

Study	Selection: 4 points				Comparability: 2 points	Exposure/outcome assessment: 3 points			Total score
	1	2	3	4	5	6	7	8	
Freitag et al. (10)	0	1	0	0	2	0	1	1	5
Rassler et al. (11)	0	1	1	1	1	0	1	1	6
Rassler et al. (34)	0	1	1	1	1	0	1	0	5
Weiner et al. (35)	0	1	1	1	1	0	1	0	5

The following items of the Newcastle-Ottawa Scale for case–control studies were included: 1. Is the case definition adequate? 2. Representativeness of the cases 3. Selection of Controls 4. Definition of Controls 5. Comparability of cases and controls on the basis of the design or analysis 6. Ascertainment of exposure 7. Same method of ascertainment for cases and controls 8. Non-Response rate. The following items of the Newcastle-Ottawa Scale for cohort studies were included: 1. Representativeness of the exposed cohort 2. Selection of the non exposed cohort 3. Ascertainment of exposure 4. Demonstration that outcome of interest was not present at start of study 5. Comparability of cohorts on the basis of the design or analysis 6. Assessment of outcome 7. Was follow-up long enough for outcomes to occur 8. Adequacy of follow up of cohorts.

Although the included studies were considered to be at least moderate quality, they faced the following methodological quality issues. Both RCTs by Huang (51) and Fregonezi et al. (52) lacked accurate information on concealed allocation, blind subjects and therapists. Huang (51) did not provide information about blind assessors, while Fregonezi et al. (52) lacked intention-to-treat analysis. Hsu et al. (9) did not clearly state whether the groups received the same measures other than the exposure or intervention of interest in a quasi-experimental study. Freitag et al. (10) conducted a case–control study, but this study did not represent of the cases, did not provide detailed information on the selection and definition of controls, and lacked the integrity of exposure data. The three cohort studies (11, 34, 35) face the following problems: non-representativeness of the exposed cohort, insufficient comparability, and the shortage of accurate data for outcome assessment. Furthermore, the cohort studies by Rassler et al. (34) in 2007 and Weiner et al. (35) lacked adequate follow-up of the cohorts.

### 3.4 Quality of evidence for included studies

The level of evidence of the included trials in this systematic review was evaluated from 2 to 4 according to the OCEBM grading of evidence. Two RCTs (51, 52) with good methodological quality and one quasi-experimental study (9) with high quality were graded level 2. One case–control study (10) with moderate quality was graded level 4. The remaining cohort studies (11, 34, 35) with moderate quality were graded level 3.

## 4 Discussion

This systematic review aimed to assess the available evidence to clarify whether RMT is effective for the treatment of respiratory and functional outcomes in patients with MG. The current comprehensive evidence supports that RMT can improve respiratory muscle endurance and functional outcomes in patients with MG. However, whether RMT can improve respiratory muscle strength and pulmonary function in patients with MG still lacks sufficient evidence, although some studies have shown that RMT can improve respiratory muscle strength and pulmonary function in patients with MG, the results of different types of studies are varied. Although currently available evidence suggests that RMT can improve respiratory muscle endurance and functional outcome in patients with MG, high-quality evidence is lacking. The studies included in this review consist of two RCTs (51, 52), one quasi-controlled study (9), one case–control study (10), and three cohort studies (11, 34, 35). However, many of these studies provide limited evidence due to a lack of reliable and high-quality research. Moreover, there was significant clinical heterogeneity among the included studies. Therefore, more rigorous RCTs are needed to study this area in the future.

The heterogeneity in outcomes regarding the impact of RMT on MIP and MEP in MG patients warrants careful interpretation (9–11, 34, 35, 52). A previous review (36) showed that RMT could improve respiratory muscles in patients with MG, but only three studies were included in that review. In our review, two of the studies (35, 52) from the previous review were included, while one study (53) was excluded because it mixed three diseases, including amyotrophic lateral sclerosis, MG, and progressive muscular disease. Our expanded analysis reveals that while improvements in respiratory muscle strength were observed in specific contexts (35, 52), the absence of significant changes in other studies (9–11, 34) likely reflects methodological and clinical variations rather than intrinsic inefficacy of RMT. Critically, studies reporting null effects consistently enrolled patients with normal or near-normal baseline respiratory muscle function (9–11, 34), inherently limiting measurable improvement potential. Conversely, trials demonstrating efficacy typically involved either combined IMT/EMT approaches (35) or patients with greater physiological deficit (52), suggesting baseline impairment severity modulates RMT responsiveness. Protocol differences constitute another key determinant. Positive outcomes correlated with longer intervention durations (8–12 weeks) (35, 52), whereas shorter regimens showed limited efficacy (9–11, 34). This aligns with established neuromuscular adaptation timelines requiring sustained stimulus (35, 52). Furthermore, the specific type of RMT significantly influences its efficacy. IMT + EMT improved MIP and MEP in patients with mild to moderate MG (35), while isolated IMT produced primarily benefits in severe cases (35), demonstrating distinct mechanistic pathways according to disease severity. Rather than invalidating RMT, these apparent contradictions highlight the intervention’s context-dependency. Therefore, more high-quality RCTs are needed in the future to further study the impact of different types of RMT intensity and treatment cycle on MG patients with different severities.

The consistent improvement in respiratory muscle endurance across studies investigating RMT in MG patients constitutes a clinically significant finding in this review (10, 11, 34, 35, 52). This aligns with conclusions from a prior systematic review in the field (36), but expands the evidence base to demonstrate efficacy across diverse training protocols, including isolated IMT and IMT + EMT. Particularly noteworthy is the observed efficacy in severe MG populations through isolated IMT (35), though this finding remains constrained by limited dedicated research in this subgroup. Therefore, more high-quality RCTs are still needed to study this direction in the future. Decreased respiratory muscle endurance is a common feature in patients with MG (9, 11). It can aggravate respiratory dysfunction, increase the risk of respiratory failure, further deteriorate the patient’s functional ability, delay the patient’s recovery, and increase the patient’s economic and psychological burden (8, 9, 20). Studies have shown that improving respiratory muscle endurance may help stabilize respiratory muscle performance and improve respiratory function, thereby avoiding corresponding dysfunction and complications (54–56). This review found that RMT can improve respiratory muscle endurance in MG patients. RMT may increase respiratory muscle endurance by inducing muscle hypertrophy and thereby improving neuromuscular coordination (11). The improvement of respiratory muscle endurance may reduce dyspnea and dysfunction in patients with MG, increase activity capacity, and improve living ability. Therefore, given the importance of RMT in improving respiratory muscle endurance in patients with MG, it is necessary to perform RMT in time for these patients in clinical treatment.

The observed improvements in FEV1 and FVC in patients with mild to moderate MG (9, 35) contrast with the absence of significant changes in these measures (10, 11, 34, 52) and PEF reported elsewhere (10, 11, 34). The divergent findings regarding the effects of RMT on spirometric parameters in MG patients likely reflect heterogeneity in both participant baseline characteristics and intervention protocols. Initially, research indicates that the pulmonary function metrics of individuals with mild to moderate MG typically do not decrease during the early phases of the condition (9, 11). Consequently, if most of the participants included have mild symptoms, the research results may be affected. Three studies included participants with normal or slightly low lung function at baseline (10, 11, 34). However, lung function parameters, like FEV1, PEF, and FVC, are based on short manoeuvres requiring maximal effort (9). These abilities may have little impact on patients with mild MG (11). Moreover, the specificity inherent in various forms of RMT may also affect the results. The beneficial impact of RMT on patients with MG may be contingent upon several factors, including the intensity, duration, device and other variables. The 7 studies reviewed exhibited variability in these parameters. Consequently, forthcoming research should aim to establish more standardized methodologies for RMT interventions and clearly defined inclusion criteria for participants to investigate further the implications of RMT on individuals with MG.

This review demonstrates that RMT improves functional outcomes of individuals with mild to moderate MG, evidenced by consistent benefits across multiple assessment scales despite methodological variations in outcome instrumentation (9–11, 34, 51). Notably, all contributing investigations employed IMT + EMT protocols (9–11, 34, 51), highlighting a critical evidence gap regarding isolated IMT effects. The deterioration of functional outcome in MG patients is related to the decline of respiratory muscle strength and endurance (9). The decline of respiratory muscle strength and endurance can affect the physical health of MG patients, aggravate dyspnea and fatigue in MG patients, thus limiting exercise capacity and affecting quality of life (11, 51). RMT enhances respiratory muscle endurance, directly improving ventilatory efficiency and functional reserve in MG. This physiological adaptation attenuates exercise-induced dyspnea and fatigue by maintaining adequate ventilation during physical activity, subsequently breaking the cycle of exertional limitation and improving functional outcomes through enhanced activity tolerance. Future research should prioritize investigating isolated IMT modalities and delineate optimal training parameters to maximize functional benefits. Standardization of outcome measures will further strengthen evidence synthesis in this emerging therapeutic domain.

While existing data indicates that RMT may positively influence the prognosis of patients with MG, it is essential to acknowledge several limitations inherent in this evidence when considering its clinical implementation. This study initially considered a meta-analysis, but due to the limited number of randomized controlled trials in this area and the differences in participant demographics, intervention details, and outcome measures collected among the included studies, it was more appropriate to construct a narrative synthesis. Moreover, the 7 included studies were of different types, lacking high-quality studies, and not all studies reported the outcomes of interest, so the total number of participants included in each variable may be small. Additionally, the included studies mainly focused on patients with mild to moderate MG, and only one study (35) focused on patients with severe MG. Patients with milder symptoms may have normal or slightly lower respiratory muscle strength or lung function indicators, which may have a certain impact on the results (9). Besides, the heterogeneity in medication regimens across included studies constitutes a major methodological limitation in our review. As detailed in the results, all participants received routine medical therapy, primarily cholinesterase inhibitors typically combined with immunosuppressants such as azathioprine, prednisone, methotrexate, or immunoglobulin. However, significant variations existed between trials regarding specific drug types, dosages, and treatment durations. Such pharmaceutical heterogeneity may confound the observed effects of RMT on functional outcomes, particularly since immunomodulators like corticosteroids directly affect muscle strength and fatigue tolerance, which represent core targets of RMT interventions. To address potential bias, the consistency of background therapy across both intervention and control groups enhances validity by minimizing confounding from differential medication use (9, 10, 51). Crucially, two studies (35, 52) implemented pharmacological stabilization protocols requiring at least 1 month of unchanged medication before RMT initiation. Additionally, stratified subgroup analyses by Freitag et al. demonstrated sustained RMT efficacy regardless of background therapy (*p* = 0.07 for interaction) (52), suggesting additive benefits rather than pharmacological masking. Despite these measures, residual confounding persists. Thus, we explicitly acknowledge medication variability as a key constraint for generalizing RMT efficacy. Future trials should mandate standardized reporting of drug regimens and prioritize recruiting patients with stable, optimized medical therapy to isolate RMT-specific effects, while pragmatic studies examining RMT-drug interactions are warranted to guide real-world implementation. Finally, due to the limited number of included studies, no publication bias assessment was performed. Therefore, more high-quality RCTs are needed in the future to explore the rehabilitation effects of different types of RMT on different types of MG patients.

## 5 Conclusion

This systematic review demonstrates that RMT may improve respiratory muscle endurance and functional outcomes in patients with MG. However, evidence regarding its effects on respiratory muscle strength and pulmonary function remains inconsistent and insufficient due to limited high-quality studies and significant clinical heterogeneity among existing research. Rigorously designed RCTs are necessary to validate these findings and explore the impact of different RMTs on different types of MG patients.

## Data Availability

The original contributions presented in the study are included in the article/supplementary material, further inquiries can be directed to the corresponding author.

## Glossary

MG: Myasthenia Gravis
PICOS: Population-Interventions-Comparison-Outcomes of interest-Study Design
RCTs: Randomised Control Trials
IG: Intervention Group
CG: Control Group
MGFA: Myasthenia Gravis Foundation of America
RMT: Respiratory Muscle Training
IMT: Inspiratory Muscle Training
EMT: Expiratory Muscle Training
MIP: Maximal Inspiratory Pressure
MEP: Maximal Expiratory Pressure
RE: Respiratory Endurance
FEV1: Forced Expiratory Volume in 1 s
PEP: Positive Expiratory Pressure
FVC: Forced Vital Capacity
QMG: Quantitative Myasthenia Gravis
MGC: Myasthenia Gravis Composite
ADL: Daily Living Ability
PEDro: the Physical Therapy Evidence Database scale
JBI: the Joanna Briggs Institute
NOS: the Newcastle–Ottawa Scale
OCEBM: the Oxford Centre for Evidence-Based Medicine
MVV: Maximal Voluntary Ventilation
VC: Vital Capacity
NR: Not reported in the source trial
N/A: Not Available
