# Supplementary material for: Effects of respiratory muscle training on respiratory function and functional outcomes in patients with myasthenia gravis: a systematic review
Source: Front Neurol. 2025 Sep 25;16:1667400. doi: 10.3389/fneur.2025.1667400 (PMC12507586; doi:10.3389/fneur.2025.1667400)
Supplement: Supplementary file 1 [file Supplementary_file_1.docx]

**Appendix 1- Database search**

| **PubMed** | |
| --- | --- |
| **Search** | **Search Terms** |
| #1 | Search: myasthenia gravis[MeSH Terms] |
| #2 | Search: ((myasthenia gravis[Text Word]) OR (MG[Text Word])) OR (generalized myasthenia gravis[Text Word]) |
| #3 | #1 OR #2 |
| #4 | Search: breathing exercises[MeSH Terms] |
| #5 | Search: ((((((((((Breathing Exercises[Text Word]) OR (respiratory strength training[Text Word])) OR (inspiratory strength training[Text Word])) OR (expiratory strength training[Text Word])) OR (respiratory muscle training[Text Word])) OR (RMT[Text Word])) OR (inspiratory muscle training[Text Word])) OR (IMT[Text Word])) OR (expiratory muscle training[Text Word])) OR (EMT[Text Word])) OR (breathing muscle training[Text Word]) |
| #6 | #4 OR #5 |
| #7 | Search: (((((((((((((((((((((respiratory function[Text Word]) OR (respiratory muscle strength[Text Word])) OR (maximum inspiratory pressure[Text Word])) OR (MIP[Text Word])) OR (maximum expiratory pressure[Text Word])) OR (MEP[Text Word])) OR (respiratory muscle endurance[Text Word])) OR (pulmonary function tests[Text Word])) OR (peak expiratory flow[Text Word])) OR (PEF[Text Word])) OR (forced expiratory volume in 1s[Text Word])) OR (FEV1[Text Word])) OR (forced vital capacity[Text Word])) OR (FVC[Text Word])) OR (functional outcome[Text Word])) OR (quantitative myasthenia gravis score[Text Word])) OR ( QMG score[Text Word])) OR (myasthenia gravis composite scale[Text Word])) OR (MGC scale[Text Word])) OR (MG score[Text Word])) OR (daily living ability[Text Word])) OR (ADL[Text Word]) |
| #8 | #3 AND # 6 AND #7 |

| **Embase** | |
| --- | --- |
| **Search** | **Search Terms** |
| #1 | ' myasthenia gravis '/exp |
| #2 | 'myasthenia gravis':ti,ab,kw OR 'mg':ti,ab,kw OR 'generalized myasthenia gravis':ti,ab,kw |
| #3 | #1 OR #2 |
| #4 | 'breathing exercise'/exp |
| #5 | 'respiratory strength training':ti,ab,kw OR 'inspiratory strength training':ti,ab,kw OR 'expiratory strength training':ti,ab,kw OR 'respiratory muscle training':ti,ab,kw OR rmt:ti,ab,kw OR 'inspiratory muscle training':ti,ab,kw OR imt:ti,ab,kw OR 'expiratory muscle training':ti,ab,kw OR emt:ti,ab,kw OR 'breathing muscle training':ti,ab,kw OR 'breathing exercises':ti,ab,kw |
| #6 | #4 OR #5 |
| #7 | 'respiratory function':ti,ab,kw OR 'respiratory muscle strength':ti,ab,kw OR 'maximum inspiratory pressure':ti,ab,kw OR mip:ti,ab,kw OR 'maximum expiratory pressure':ti,ab,kw OR mep:ti,ab,kw OR 'respiratory muscle endurance':ti,ab,kw OR 'pulmonary function tests':ti,ab,kw OR 'peak expiratory flow':ti,ab,kw OR pef:ti,ab,kw OR 'forced expiratory volume in 1s':ti,ab,kw OR fev1:ti,ab,kw OR 'forced vital capacity':ti,ab,kw OR fvc:ti,ab,kw OR 'functional outcome':ti,ab,kw OR 'quantitative myasthenia gravis score':ti,ab,kw OR qmg score:ti,ab,kw OR 'myasthenia gravis composite scale':ti,ab,kw OR mgc scale:ti,ab,kw OR 'mg score':ti,ab,kw OR ' daily living ability ':ti,ab,kw OR adl:ti,ab,kw |
| #8 | #3 AND # 6 AND #7 |

| **Allied and Complementary Medicine Database** | |
| --- | --- |
| **Search** | **Search Terms** |
| S1 | KW myasthenia gravis OR KW MG OR KW generalized myasthenia gravis |
| S2 | KW respiratory strength training OR KW inspiratory strength training OR KW expiratory strength training OR KW respiratory muscle training OR KW RMT OR KW inspiratory muscle training OR KW IMT OR KW expiratory muscle training OR KW EMT OR KW breathing muscle training OR KW breathing exercises |
| S3 | KW respiratory function OR KW respiratory muscle strength OR KW maximum inspiratory pressure OR KW MIP OR KW maximum expiratory pressure OR KW MEP OR KW respiratory muscle endurance OR KW pulmonary function testing OR KW peak expiratory flow OR KW PEF OR KW forced expiratory volume in 1s OR KW FEV1 OR KW forced vital capacity OR KW FVC OR KW functional outcome OR KW quantitative myasthenia gravis score OR KW QMG score OR KW myasthenia gravis composite scale OR KW MGC scale OR KW MG score OR KW daily living ability OR KW ADL |
| S4 | S1 AND S2 AND S3 |

| **Cumulative Index to Nursing and Allied Health Literature** | |
| --- | --- |
| **Search** | **Search Terms** |
| S1 | MH myasthenia gravis OR MH MG OR MH generalized myasthenia gravis |
| S2 | MH respiratory strength training OR MH inspiratory strength training OR MH expiratory strength training OR MH respiratory muscle training OR MH RMT OR MH inspiratory muscle training OR MH IMT OR MH expiratory muscle training OR MH EMT OR MH breathing muscle training OR MH breathing exercises |
| S3 | MH respiratory function OR MH respiratory muscle strength OR MH maximum inspiratory pressure OR MH MIP OR MH maximum expiratory pressure OR MH MEP OR MH respiratory muscle endurance OR MH pulmonary function testing OR MH peak expiratory flow OR MH PEF OR MH forced expiratory volume in 1s OR MH FEV1 OR MH forced vital capacity OR MH FVC OR MH functional outcome OR MH quantitative myasthenia gravis score OR MH QMG score OR MH myasthenia gravis composite scale OR MH MGC scale OR MH MG score OR MH daily living ability OR MH ADL |
| S4 | S1 AND S2 AND S3 |

| **Cochrane Library** | |
| --- | --- |
| **Search** | **Search Terms** |
| #1 | MeSH descriptor: myasthenia gravis |
| #2 | (myasthenia gravis OR MG OR generalized myasthenia gravis):ab,ti,kw |
| #3 | #1 OR #2 |
| #4 | MeSH descriptor: breathing exercises |
| #5 | (respiratory strength training OR inspiratory strength training OR expiratory strength training OR respiratory muscle training OR RMT OR inspiratory muscle training OR IMT OR expiratory muscle training OR EMT OR breathing muscle training OR breathing exercises):ab,ti,kw |
| #6 | #4 OR #5 |
| #7 | (respiratory function OR respiratory muscle strength OR maximum inspiratory pressure OR MIP OR maximum expiratory pressure OR MEP OR respiratory muscle endurance OR pulmonary function testing OR peak expiratory flow OR PEF OR forced expiratory volume in 1s OR FEV1 OR forced vital capacity OR FVC OR functional outcome OR quantitative myasthenia gravis score OR QMG score OR myasthenia gravis composite scale OR MGC scale OR MG score OR daily living ability OR ADL):ab,ti,kw |
| #8 | #3 AND # 6 AND #7 |

| **China National Knowledge Infrastructure databases (English version)** | |
| --- | --- |
| **Search** | **Search Terms** |
| S1 | (Keyword: myasthenia gravis) OR (Keyword: MG) OR (Keyword：generalized myasthenia gravis) |
| S2 | (Keyword：respiratory strength training) OR (Keyword：inspiratory strength training) OR (Keyword：expiratory strength training) OR (Keyword：respiratory muscle training) OR (Keyword: RMT) OR (Keyword：inspiratory muscle training) OR (Keyword：IMT) OR (Keyword: expiratory muscle training) OR (Keyword：EMT) OR (Keyword: breathing muscle training) OR (Keyword：breathing Exercises) |
| S3 | (Keyword: respiratory function) OR (Keyword: respiratory muscle strength) OR (Keyword: maximum inspiratory pressure) OR (Keyword: MIP) OR (Keyword: maximum expiratory pressure) OR (Keyword: MEP) OR (Keyword: respiratory muscle endurance) OR (Keyword: pulmonary function tests) OR (Keyword: peak expiratory flow) OR (Keyword: PEF) OR (Keyword: forced expiratory volume in 1s) OR (Keyword: FEV1) OR (Keyword: forced vital capacity) OR (Keyword: FVC) OR (Keyword: functional outcome) OR (Keyword: quantitative myasthenia gravis score) OR (Keyword: QMG score) OR (Keyword: myasthenia gravis composite scale) OR (Keyword: MGC scale) OR (Keyword: MG score) OR (Keyword: daily living ability) OR (Keyword: ADL) |
| S4 | S1 AND S 2 AND S3 |

| **China National Knowledge Infrastructure databases (Chinese version)** | |
| --- | --- |
| **检索** | **检索术语** |
| #1 | (关键词: 重症肌无力) OR (关键词: MG) OR (关键词：全身性重症肌无力) |
| #2 | (关键词：呼吸力量训练) OR (关键词：吸气力量训练) OR (关键词：呼气力量训练) OR (关键词：呼吸肌训练) OR (关键词: RMT) OR (关键词：吸气肌训练) OR (关键词：IMT) OR (关键词: 呼气肌训练) OR (关键词：EMT) OR (关键词: 呼吸肌肉训练) OR (关键词：呼吸训练) |
| #3 | (关键词: 呼吸功能) OR (关键词: 呼吸肌力量) OR (关键词: 最大吸气压) OR (关键词: MIP) OR (关键词: 最大呼气压) OR (关键词: MEP) OR (关键词: 呼吸肌耐力) OR (关键词: 肺功能测试) OR (关键词: 呼气峰值流量) OR (关键词: PEF) OR (关键词: 1秒内用力呼气量) OR (关键词: FEV1) OR (关键词: 用力肺活量) OR (关键词: FVC) OR (关键词:功能结局) OR (关键词: 重症肌无力定量评分) OR (关键词: QMG score) OR (关键词: 重症肌无力综合量表) OR (关键词: MGC scale) OR (关键词: MG score) OR (关键词:日常生活能力) OR (关键词: ADL) |
| #4 | #1 AND # 2 AND #3 |
